# Prospective external validation of the Predicting Out-of-OFfice Blood Pressure (PROOF-BP) strategy for triaging ambulatory monitoring in the diagnosis and management of hypertension: observational cohort study

**DOI:** 10.1136/bmj.k2478

**Published:** 2018-06-27

**Authors:** James P Sheppard, Una Martin, Paramjit Gill, Richard Stevens, FD Richard Hobbs, Jonathan Mant, Marshall Godwin, Janet Hanley, Brian McKinstry, Martin Myers, David Nunan, Richard J McManus

**Affiliations:** 1 Nuffield Department of Primary Care Health Sciences, University of Oxford, Radcliffe Primary Care, OX2 6GG Oxford, UK; 2 Institute of Clinical Sciences, University of Birmingham, Birmingham, UK; 3 Warwick Medical School, University of Warwick, Coventry, UK; 4 University of Cambridge, Cambridge, UK; 5 Memorial University of Newfoundland, St John’s, Canada; 6 Edinburgh Napier University, Edinburgh, UK; 7 University of Edinburgh, Edinburgh, UK; 8 University of Toronto, Toronto, Canada

## Abstract

**Objective:**

To prospectively validate the Predicting Out-of-OFfice Blood Pressure (PROOF-BP) algorithm to triage patients with suspected high blood pressure for ambulatory blood pressure monitoring (ABPM) in routine clinical practice.

**Design:**

Prospective observational cohort study.

**Setting:**

10 primary care practices and one hospital in the UK.

**Participants:**

887 consecutive patients aged 18 years or more referred for ABPM in routine clinical practice. All underwent ABPM and had the PROOF-BP applied.

**Main outcome measures:**

The main outcome was the proportion of participants whose hypertensive status was correctly classified using the triaging strategy compared with the reference standard of daytime ABPM. Secondary outcomes were the sensitivity, specificity, and area under the receiver operator characteristic curve (AUROC) for detecting hypertension.

**Results:**

The mean age of participants was 52.8 (16.2) years. The triaging strategy correctly classified hypertensive status in 801 of the 887 participants (90%, 95% confidence interval 88% to 92%) and had a sensitivity of 97% (95% confidence interval 96% to 98%) and specificity of 76% (95% confidence interval 71% to 81%) for hypertension. The AUROC was 0.86 (95% confidence interval 0.84 to 0.89). Use of triaging, rather than uniform referral for ABPM in routine practice, would have resulted in 435 patients (49%, 46% to 52%) being referred for ABPM and the remainder managed on the basis of their clinic measurements. Of these, 69 (8%, 6% to 10%) would have received treatment deemed unnecessary had they received ABPM.

**Conclusions:**

In a population of patients referred for ABPM, this new triaging approach accurately classified hypertensive status for most, with half the utilisation of ABPM compared with usual care. This triaging strategy can therefore be recommended for diagnosis or management of hypertension in patients where ABPM is being considered, particularly in settings with limited resources.

## Introduction

Hypertension is an important risk factor for cardiovascular disease,^1^ the leading cause of morbidity and mortality worldwide.^2^ It can be managed effectively with antihypertensive drugs.^3^ Ambulatory blood pressure monitoring (ABPM) is the reference standard for confirming a diagnosis of hypertension and is now commonly used before initiation of treatment.^3^ This is because ambulatory blood pressure has been shown to estimate mean blood pressure more accurately and to correlate better with a range of cardiovascular outcomes than blood pressure measured in a clinic setting.^4^ ^5^ ^6^ Indeed, clinic blood pressure often misclassifies true mean blood pressure owing to white coat hypertension (high clinic blood pressure with normal ambulatory blood pressure) or the converse, masked hypertension (high ambulatory blood pressure with normal clinic blood pressure; see supplementary eFigure 1).^7^ ^8^ ^9^

Current strategies for the diagnosis of hypertension recommended by the UK National Institute for Health and Care Excellence and the American College of Cardiology/American Heart Association Task Force state that to confirm a diagnosis of hypertension people should undergo out-of-office measurement (ambulatory or home blood pressure monitoring) if blood pressure is raised in the clinic.^10^ ^11^ This method is cost effective as it reduces misdiagnosis caused by white coat hypertension.^3^ However, this approach is not perfect since in addition to detecting white coat hypertension, it results in other patients with true underlying hypertension identified by clinic blood pressure readings being sent for unnecessary out-of-office monitoring. Additionally it will not capture those patients with masked hypertension and is not currently used routinely in treated patients with uncontrolled hypertension who might also be misclassified owing to white coat or masked effects.

Triaging patients for ABPM has been suggested to improve the management of hypertension by limiting the use of ABPM to those most likely to be misclassified by clinic blood pressure measurements.^12^ The most effective way to triage patients for ABPM has been debated,^13^ ^14^ but recent work proposes an individualised triaging approach using multiple clinic readings and patient characteristics (eFigures 2 and 3).^12^ ^15^

This approach, using the algorithm Predicting Out-of-OFfice Blood Pressure (PROOF-BP), has been validated^12^ and seems to be cost effective in a research setting,^16^ but blood pressure measurements taken under such controlled conditions are not necessarily comparable to blood pressure readings in routine clinical practice.^17^ ^18^ ^19^ Differences can occur due to suboptimal measurement techniques^20^ ^21^ ^22^ and rounding bias (rounding off readings to the nearest zero value).^23^ ^24^ Thus, a triaging approach shown to be accurate in a research setting might not be as precise in routine clinical practice. We collected relevant data to prospectively validate this new triaging approach in routine clinical practice in both primary and secondary care.

## Methods

The protocol for this prospective, multicentre observational cohort study has been published previously.^25^ The supplementary appendix provides detailed methods and the prespecified analysis plan.

### Study participants and setting

Between May 2015 and January 2017 we enrolled consecutive patients attending participating centres in both primary and secondary care, for whom ABPM was considered appropriate. Eligible participants were those undergoing ABPM as a result of routine blood pressure screening or monitoring in primary care or through referral to secondary care with suspected hypertension, newly diagnosed or treated hypertension, resistant hypertension, secondary hypertension, or other conditions requiring specialist advice. Anonymised data were collected on all patients fulfilling the eligibility criteria. We excluded patients if they lacked basic clinical information, did not have multiple clinic blood pressure readings recorded (obtained on at least three occasions within the same visit), and did not wear the ambulatory blood pressure monitor as instructed.

### Procedures

All participants underwent ABPM, clinic blood pressure measurement, and collection of patient characteristics. The protocol provides information on the data collected for each participant,^25^ which included blood pressure measurements (values and measurement technique), previous treatment prescriptions, body mass index, smoking status, and history of diabetes, chronic kidney disease, atrial fibrillation, and cardiovascular disease. Changes to antihypertensive treatment after ABPM were not recorded. All data were collected from electronic health records and trained staff at each data collection site entered data directly onto the study database.

To capture as close to routine blood pressure measurements as possible, staff at participating sites were asked to measure clinic and ambulatory blood pressure, but we did not suggest or recommend a specific protocol for measurement. We defined routine blood pressure as readings taken by the consulting healthcare professional as part of standard clinical practice.

For participants to be included in the study, we required a minimum of three clinic readings taken at the time of referral for ABPM or at fitting of the blood pressure monitor. Even though three measurements are recommended in guidelines,^10^ this may not always occur in routine practice.^26^ We specified three readings to be taken as mandatory to permit validation of the triaging algorithm. We offered each site a validated automated blood pressure monitoring device (Omron M10-IT; Omron, Kyoto, Japan) to assist with the collection of multiple clinic blood pressure readings, but with the option to continue with use of their own monitor, so long as at least three readings were taken and recorded. To our knowledge, all readings were taken with the doctor or nurse present. ABPM was conducted using the practice or hospital’s own ambulatory monitor and fitted by a trained nurse or allied health professional. Some practices in primary care only collected daytime ambulatory pressures. Supplementary eTable 1 gives details of clinic and ambulatory blood pressure monitors used at each site.

### The PROOF-BP triaging approach

The triaging strategy applied an algorithm to three blood pressure readings taken at the clinic appointment, combined with information from an individual’s electronic health record: age, sex, body mass index, hypertensive and treatment history, and the presence of cardiovascular disease (eFigures 2 and 3).^12^ This algorithm identified three groups: those with definitively normal blood pressure, those with definitively high blood pressure, and those requiring further investigation using ABPM (see methods in online appendix for more detail).

### Primary outcome

The primary outcome was the proportion of participants whose hypertensive status was correctly classified using the triaging strategy compared with the reference standard of daytime ABPM (using a threshold for hypertension of ≥135/85 mm Hg).^10^ ^11^ ^27^ This was defined as the proportion of patients with sustained hypertension (true positives), normotension (true negatives), white coat hypertension (false positives), and masked hypertension (false negatives).

### Secondary outcomes

We estimated the sensitivity (for detecting hypertension in those with the condition), specificity (for ruling out hypertension in those without the condition), and positive and negative predictor values, and we compared these with guideline strategies for measuring blood pressure from the United Kingdom,^10^ United States,^11^ Europe,^27^ Canada,^28^ and Japan^29^ (eTable 2). Further secondary outcomes included accuracy of the triaging strategy in different subgroups: setting (primary care *v* secondary care), age (<65 years *v* ≥65 years), sex, smoking status (never or former smoker *v* current smoker), body mass index (<30 kg/m^2^ *v* ≥30 kg/m^2^), and history of hypertension, diabetes, chronic kidney disease, and cardiovascular disease.

### Data analysis

We used descriptive statistics to describe the number of patients classified with sustained hypertension, white coat hypertension, normotension, and masked hypertension with the triaging approach (the primary outcome) compared with daytime ABPM as the reference standard. These were used to calculate the sensitivity, specificity, and positive and negative predictor values of the triaging approach, and the total proportion of participants with correctly classified hypertensive status and proportion that would have been referred for ABPM.

To examine model performance, we constructed a logistic regression model with true hypertension (defined by daytime ABPM) as the dependant outcome variable and classification using the triaging approach as the independent predictor variable. From this model we estimated the area under the receiver operating characteristic (AUROC) curve statistic. Further analyses were conducted examining the primary outcomes using different definitions of clinic and ambulatory blood pressure (see online appendix).

### Post hoc analyses

Post hoc analyses were undertaken to examine performance of the PROOF-BP algorithm on its own (without additional ABPM) and this was compared with other blood pressure measurement strategies (employed without ABPM).^10^ ^11^ ^30^ ^31^ ^32^ Subgroup analyses of the sensitivity and specificity of the PROOF-BP triaging approach were undertaken and accompanied by one additional non-prespecified subgroup: patients in whom the treating clinician’s own monitor was used to measure clinic blood pressure compared with those where monitors were provided by the research team.

All analyses were conducted using STATA version 13.1 (MP parallel edition, StataCorp, TX). Results are presented as means or proportions, with standard deviations or 95% confidence intervals, unless stated otherwise.

### Sample size

Based on the original validation of the PROOF-BP prediction model,^12^ accrual of data from at least 800 patients was required for estimation of hypertensive status with an accuracy to within 2-6% (see eTable 3).^25^ To ensure that the prespecified subgroup analysis could be adequately powered we specified a sample size of up to 1000 patients.

### Patient and public involvement

Patients with a history of hypertension were approached to discuss the study at the design phase of the project. In particular their opinions were sought on the methods of recruitment and patient facing study literature, before ethical and NHS R&D applications.

## Results

### Recruitment and baseline population

**Fig 1:**
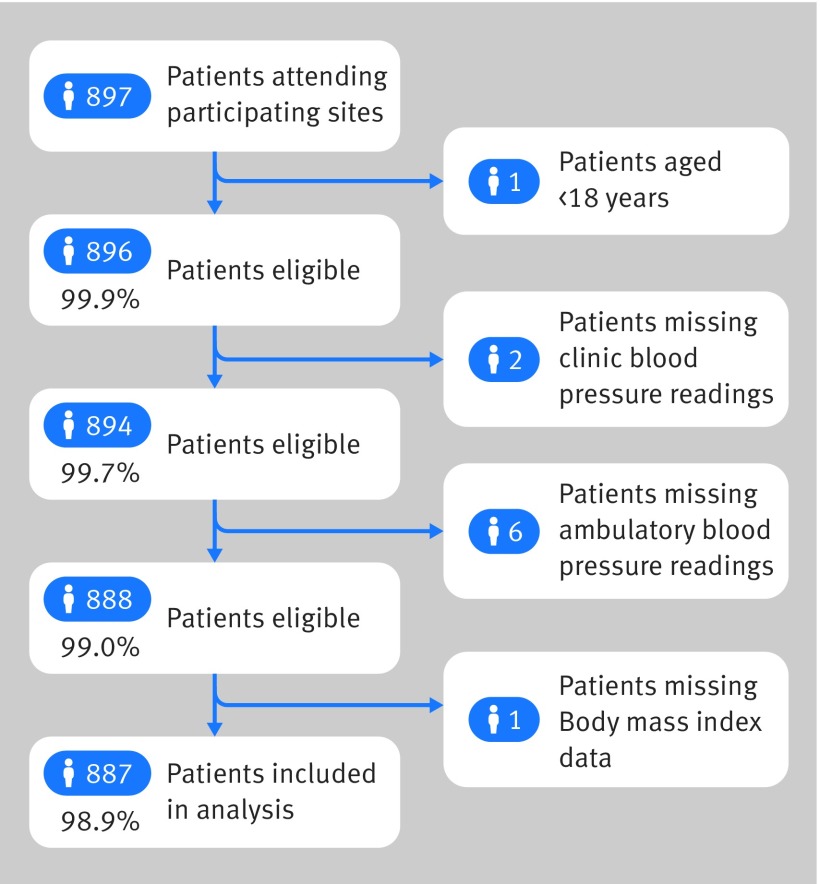
Patient recruitment flow diagram

Ten general practice surgeries and one hospital trust participated in the study. A total of 897 patients attended participating sites for clinic blood pressure measurement and ABPM during the study period. In total, 10 patients (1.1%) were ineligible: eight had missing blood pressure readings, one was aged less than 18 years, and one had missing clinical information (fig 1). The remaining patients (n=887, 99%) were enrolled into the study and included in the primary analysis.

Mean age was 52.8 (SD 16.2) years, 53.8% were women, and 14.0% were current smokers (table 1). Mean clinic blood pressure at referral for ABPM was 147/91 (SD 19/13) mm Hg. A small proportion had diabetes, chronic kidney disease, and a history of cardiovascular disease. Approximately 70% had a history of hypertension and 61% of the total population were taking at least one antihypertensive drug. Patients attending secondary care were younger and had higher blood pressure, more comorbidities, and were taking more antihypertensives than those attending primary care (table 1). Most patients were referred for ABPM owing to suspected hypertension or uncontrolled blood pressure (eTable 4).

**Table 1:** Baseline characteristics

Characteristics	All patients (n=887)	Primary care (n=354)	Secondary care (n=533)
Mean (SD) age (years)	52.8 (16.2)	57.4 (16.2)	49.8 (16.9)
Sex (% women)	477 (53.8)	184 (52.0)	293 (55.0)
Ethnicity:			
White	637 (71.8)	286 (80.8)	351 (65.9)
Black	67 (7.6)	16 (4.5)	51 (9.6)
South Asian	114 (12.9)	34 (9.6)	80 (15.0)
Other	28 (3.2)	2 (0.6)	26 (4.9)
Unknown	41 (4.6)	16 (4.5)	41 (4.6)
Smoking status:			
Current	124 (14.0)	55 (15.5)	69 (13.0)
Former	205 (23.1)	113 (31.9)	92 (17.3)
Never	499 (56.3)	181 (51.1)	318 (59.7)
Unknown	59 (6.7)	5 (1.4)	54 (10.1)
Mean (SD) body mass index	30.8 (8.1)	30.3 (7.2)	31.1 (8.6)
Diabetes	116 (13.1)	46 (13.0)	70 (13.1)
Chronic kidney disease (stages 3-5)	59 (6.7)	20 (5.7)	39 (7.3)
Atrial fibrillation*	28 (3.3)	15 (4.3)	13 (2.6)
Diagnosis of hypertension	619 (69.8)	141 (39.8)	478 (89.7)
Cardiovascular drugs*:			
ACE inhibitor	265 (30.1)	51 (14.5)	214 (40.4)
Angiotensin II receptor blocker	129 (14.6)	24 (6.9)	105 (19.8)
Calcium channel blocker	279 (31.6)	52 (14.9)	227 (42.8)
Thiazide	170 (19.3)	24 (6.9)	146 (27.5)
β blocker	138 (15.7)	32 (9.1)	106 (19.9)
α blocker	92 (10.4)	16 (4.6)	76 (14.3)
Other antihypertensive	45 (5.1)	4 (1.1)	41 (7.7)
Statin	234 (26.7)	114 (32.7)	120 (22.7)
Antiplatelet	118 (13.4)	47 (13.5)	71 (13.4)
No of antihypertensives:			
0	349 (39.4)	239 (67.5)	110 (20.6)
1	217 (24.5)	57 (16.1)	160 (30.0)
2	149 (16.8)	36 (10.2)	113 (21.2)
3	102 (11.5)	17 (4.8)	85 (16.0)
≥4	70 (7.9)	5 (1.4)	65 (12.2)
Medical history:			
Coronary heart disease	59 (7.1)	20 (5.7)	39 (8.1)
Stroke or transient ischaemic attack	41 (4.9)	15 (4.3)	26 (5.3)
Heart failure	11 (1.3)	0 (0)	11 (2.2)
Peripheral vascular disease	12 (1.4)	5 (1.4)	7 (1.4)
Mean (SD) blood pressure readings (mm Hg):			
Clinic systolic	146.7 (19.2)	143.8 (17.5)	148.7 (20.1)
Clinic diastolic	90.6 (12.5)	89.3 (10.0)	91.5 (13.8)
Daytime systolic ABPM	140.8 (16.8)	140.2 (17.8)	141.2 (16.2)
Daytime diastolic ABPM	83.4 (11.7)	81.9 (11.6)	85.3 (11.5)
Night time systolic ABPM	128.5 (19.5)	125.1 (18.0)	130.0 (18.8)
Night time diastolic ABPM	74.0 (12.0)	70.7 (11.0)	75.7 (11.8)
24 hour systolic ABPM	137.5 (17.0)	135.0 (16.6)	138.2 (16.1)
24 hour diastolic ABPM	81.4 (11.3)	78.6 (10.9)	83.0 (11.1)
Type of hypertension at visits (1st reading):			
Clinic	695 (78.4)	268 (75.7)	427 (80.1)
White coat	173 (19.5)	87 (24.6)	86 (16.1)
Masked	79 (8.9)	36 (10.2)	43 (8.1)

ACE=angiotensin converting enzyme; ABPM=ambulatory blood pressure monitoring.

* Proportion in those with available data.

### Primary analysis

The triaging strategy (algorithm used in combination with ABPM) predicted true blood pressure (true positives 66%, 95% confidence interval 63% to 69%; true negatives 24%, 22% to 27%) with a low error rate (false positives 8%, 6% to 10%; false negatives 2%, 1% to 3%) (table 2). The triaging strategy resulted in 49% (46% to 52%) being referred for ABPM and the remainder managed on the basis of their clinic measurements. Of the latter, 69 (8%, 6% to 10%) would have received treatment that would have been deemed unnecessary had they received an ABPM (table 2).

**Table 2:** Classification of patients and utilisation of ABPM using the triaging approach. Values are number of participants; percentage (95% confidence interval) unless stated otherwise

Population	No (%) of total population	True positive (sustained hypertensive)	True negative (normotensive)	False positive (white coat hypertensive)	False negative (masked hypertensive)	Correctly classified	Utilisation of ABPM
Overall	887 (100)	584; 66 (63 to 69)	217; 24 (22 to 27)	69; 8 (6 to 10)	17; 2 (1 to 3)	801; 90 (88 to 92)	435; 49 (46 to 52)
For diagnosis*	268 (30)	156; 58 (52 to 64)	88; 33 (27 to 39)	16; 6 (3 to 10)	8; 3 (1 to 6)	244; 91 (87 to 94)	165; 62 (55 to 67)
For management†	619 (70)	428; 69 (65 to 73)	129; 21 (18 to 24)	53; 9 (6 to 11)	9; 1 (0.7 to 3)	557; 90 (87 to 92)	270; 44 (40 to 48)

ABPM=ambulatory blood pressure monitoring.

* Patients without a history of hypertension.

† Patients with a history of hypertension.

### Secondary analysis

The triaging strategy had a sensitivity of 97% (95% confidence interval 96% to 98%) and specificity of 76% (95% confidence interval 71% to 81%), and an AUROC of 0.86 (95% confidence interval 0.84 to 0.89) for predicting true hypertension (table 3). It would have resulted in higher sensitivity and negative predictive values but lower specificity and positive predictive values compared with other diagnostic strategies had they been applied to this population (including that recommended in the UK (eTables 5 and 6).

**Table 3:** Accuracy of triaging approach. Values are percentages (95% confidence intervals) unless stated otherwise

Population	Total population	AUROC (95% CI)	Sensitivity	Specificity	Positive predictive value	Negative predictive value
Overall	887 (100)	0.86 (0.84 to 0.89)	97.2 (95.8 to 98.5)	75.9 (70.9 to 80.8)	89.4 (87.1 to 91.8)	92.7 (89.4 to 96.1)
For diagnosis*	268 (30)	0.90 (0.86 to 0.94)	95.1 (91.8 to 98.4)	84.6 (77.7 to 91.5)	90.7 (86.4 to 95.0)	91.7 (86.1 to 97.2)
For management†	619 (70)	0.84 (0.81 to 0.88)	97.9 (96.6 to 99.3)	70.9 (64.3 to 77.5)	89.0 (86.2 to 91.8)	93.5 (89.4 to 97.6)

AUROC=area under the receiver operating characteristic curve.

* Patients without a history of hypertension.

† Patients with a history of hypertension.

**Fig 2:**
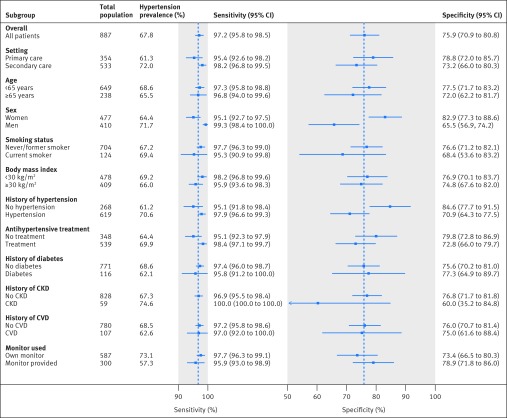
Post hoc analyses examining sensitivity and specificity of PROOF-BP triaging strategy for hypertension, by subgroups. CKD=chronic kidney disease; CVD=cardiovascular disease

**Fig 3:**
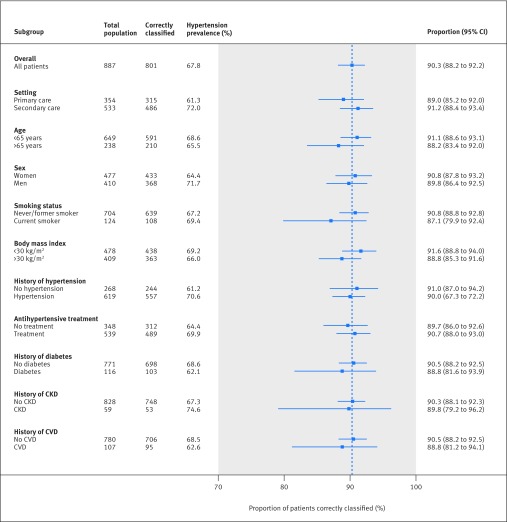
Proportion of patients correctly classified with hypertension using PROOF-BP triaging strategy, by subgroups. CKD=chronic kidney disease; CVD=cardiovascular disease

**Fig 4:**
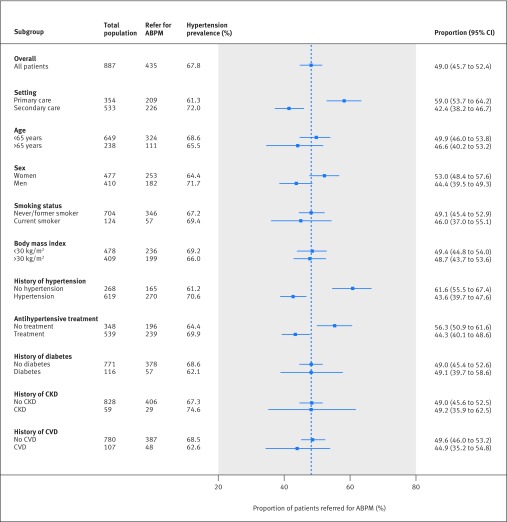
Proportion of patients referred for ambulatory blood pressure monitoring (ABPM) using PROOF-BP triaging strategy, by subgroups. CKD=chronic kidney disease; CVD=cardiovascular disease

The sensitivity of the triaging strategy was consistently high (95-100%) regardless of the setting, underlying prevalence of hypertension, subgroup population, or clinic blood pressure monitor used (table 3, fig 2). Specificity was affected by the population, with values as low as 60% in patients with chronic kidney disease and as high as 85% in patients with no previous diagnosis of hypertension (fig 2). Hypertensive status was correctly classified in 87-91% of patients, regardless of the setting, patient characteristics, comorbidities, or history of hypertension or treatment (fig 3). However, a lower prevalence of hypertension with more individuals with intermediate blood pressures resulted in more referrals for ABPM in a primary care setting (59% *v* 42% secondary care) and in those patients with no history of hypertension (62% *v* 44% history of hypertension) or prescription for antihypertensives (56% *v* 44% existing prescription) (fig 4).

The triaging approach performed consistently well in terms of hypertensive classification and lower utilisation of ABPM, regardless of the number of clinic blood pressure readings or period of 24 hours used to define ambulatory blood pressure (eTable 7). The approach did result in fewer patients being correctly classified according to night time blood pressure (90.3% primary analysis *v*. 87.5% night time readings), reflecting the different diagnostic thresholds used to define night time hypertension. The performance of the PROOF-BP algorithm was reduced when utilised without ABPM—that is, not as a triaging tool (eTable 8).

## Discussion

This study aimed to prospectively validate a new approach to determine who should be offered ambulatory blood pressure monitoring (ABPM) for suspected or poor control of hypertension in real world settings. In a population being referred for ABPM, use of a triaging strategy captured nearly all patients with hypertension while resulting in half the number of referrals for resource intensive ABPM compared with usual care. However, such a strategy would misclassify as hypertensive, one in four of those without hypertension, potentially leading to unnecessary treatment. Used in a diagnostic situation in people without a previous diagnosis of hypertension, this misclassification of normotensive people was reduced to one in seven. These findings suggest the potential for diagnosing and managing hypertension using a triaging strategy to reduce the need for ABPM.

### Strengths and weaknesses of this study

This study was conducted according to a prepublished, peer reviewed protocol, with analyses undertaken in line with a prespecified analysis plan. The study included 98.8% of patients attending routine practice during the study period, suggesting it is likely to be representative of the target population (those who have an indication for ABPM) and was sufficiently powered to examine key outcomes.

Unlike the previous studies in which the PROOF-BP triaging algorithm has been previously validated,^12^ ^30^ ^31^ ^32^ ^33^ the measurement of clinic blood pressure was not standardised in terms of requiring healthcare professionals to use certain monitors or to conform to a specific measurement protocol. This was deliberate in an attempt to replicate readings taken in routine clinical practice where techniques for blood pressure measurement can vary.^34^ It is recognised that such readings may not truly reflect routine practice either, since healthcare professionals knew they were participating in a research study and validated monitors were provided to some sites to aid the capture of three consecutive clinic readings.^32^ In total, six of the 11 participating sites used the validated clinic blood pressure monitors supplied by the research team, but this had no impact on the performance of the triaging approach.

The study included patients in whom the treating doctor felt ABPM was appropriate. In most cases this was owing to suspected hypertension or apparently uncontrolled blood pressure (eTable 4). This included patients with no history of hypertension (ie, those referred for diagnosis) and those already prescribed blood pressure lowering drugs (ie, those referred for management of hypertension). The findings are therefore widely applicable to patients undergoing diagnosis and also applicable to those in whom the consulting doctor is considering treatment intensification but is unsure if this is appropriate. Although additional monitoring is not recommended in those with normal blood pressure,^10^ ^11^ ^27^ 22% of the present cohort had clinic readings below 140/90 mm Hg before ABPM and 9% proved to have masked hypertension, which is consistent with previous prevalence estimates.^35^ ^36^ ^37^

### Comparison with other literature

Few studies have previously identified an effective method for triaging patients for out-of-office blood pressure monitoring, and none have externally validated such a method using data from a routine clinical setting. The original PROOF-BP derivation paper reported good performance of the algorithm when used in combination with ABPM, with a sensitivity of 96% and specificity of 87%. One study examined the optimal threshold for referral for out-of-office monitoring based on automated clinic blood pressure levels in patients with normal clinic pressure for detection of masked hypertension.^13^ The authors reported a sensitivity of 76% and specificity of 58% for a clinic blood pressure threshold of 120 mm Hg systolic. In practice, such a threshold would have limited accuracy and efficiency, missing one in four patients with hypertension and resulting in a large number of patients being referred for ABPM. As such, they did not recommend this approach for use in routine clinical practice. The PROOF-BP triaging strategy examined here uses an individualised approach, taking into account a patient’s clinic blood pressure level and variability and underlying cardiovascular risk. It is an approach that maximises the accuracy of hypertensive classification while minimising ABPM use compared with usual care, which uses fixed thresholds for referral in all patients.

### Implications for clinical practice

Use of a triaging approach would substantially reduce the proportion of people requiring ABPM at the expense of “over treating” a small proportion of individuals. The triaging algorithm performed consistently across sensitivity analyses and subgroup analyses. Because the triaging mechanism is based on thresholds of adjusted clinic blood pressure, scenarios where a greater number of individuals have blood pressures close to the diagnostic threshold for hypertension are likely to result in greater numbers of patients being referred for ABPM. The greater the use of ABPM, the more accurate the triaging strategy was (in patients with no history of hypertension the sensitivity was 95% and specificity 85%, but 61% were referred for ABPM). Where ABPM facilities are widely available, clinicians may therefore want to consider additional use of ABPM, where the risk of misdiagnosis is great but an individual is not within the triaging range. This could include younger, lower risk people for whom a lifetime of treatment may have a larger impact on their quality of life. However, in low and middle income countries where resources might be more stretched, the PROOF-BP triaging approach represents an alternative option that maximises the benefits of ABPM and targets those with the most to gain.

These data suggest that the PROOF-BP triaging strategy may be used in both primary and secondary care wherever ABPM is being considered to rule out white coat hypertension. To facilitate uptake, the algorithm is freely available as a calculator online (https://sentry.phc.ox.ac.uk/proof-bp) and could be incorporated into clinic computer systems, smartphone blood pressure management apps, and Bluetooth enabled monitors, providing general practitioners and hospital doctors with instant feedback and management recommendations in terms of referral or treatment.

### Conclusions

This prospective, external validation study shows the accuracy of the PROOF-BP algorithm as a tool for triaging patients for ABPM in routine clinical practice. Used in conjunction with ABPM, the algorithm identifies most patients with hypertension and results in half as many patients being referred for additional ABPM compared with usual care. It may, however, lead to a small number of patients receiving unnecessary treatment. Such an approach can now be recommended for use in both primary and secondary care, wherever ABPM is being considered to rule out white coat hypertension or masked hypertension.

**What is already known on this topic**

Daytime ambulatory blood pressure monitoring (ABPM) is the recognised standard for measuring blood pressure and diagnosing hypertension
ABPM is currently recommended for all patients with raised screening clinic pressure, but triaging patients for this additional monitoring may be a more effective strategy
Blood pressure readings taken in research studies are not always comparable to those taken in clinical practice where there may be suboptimal measurement technique and rounding bias

**What this study adds**

The PRedicting Out-of-OFfice Blood Pressure (PROOF-BP) algorithm accurately classified hypertensive status with half the utilisation of ABPM compared with usual care
The triaging strategy resulted in 435 participants (49%, 95% confidence interval 46% to 52%) being referred for ABPM and the remainder managed on the basis of their clinic measurements; 69/435 (8%, 6% to 10%) would have received treatment deemed unnecessary had they received ABPM
This strategy can be used to triage for ABPM in both a primary and secondary care and for diagnosis or management of hypertension

## Web extra

Extra material supplied by authors

**Supplementary information:** figures 1-3, extended methods, tables 1-8, statistical analysis plan, and references

## Notes

Ethical approval: This study was approved by the National Research Ethics Service Committee South Central–Oxford A (reference 15/SC/0184), and site specific research and development approval acquired from the relevant NHS trusts.

Data sharing: Anonymised, patient level data are available from the corresponding author upon request, subject to submission of a suitable study protocol and analysis plan.

Transparency: The manuscript’s guarantor (JPS) affirms that the manuscript is an honest, accurate, and transparent account of the study being reported; that no important aspects of the study have been omitted; and that any discrepancies from the study as planned have been explained.
