# Supplementary material for: Prospective external validation of the Predicting Out-of-OFfice Blood Pressure (PROOF-BP) strategy for triaging ambulatory monitoring in the diagnosis and management of hypertension: observational cohort study
Source: BMJ. 2018 Jun 27;361:k2478. doi: 10.1136/bmj.k2478 (PMC6020747; doi:10.1136/bmj.k2478)
Supplement: Supplementary file 1 — Supplementary information: figures 1-3, extended methods, tables 1-8, statistical analysis plan, and references [file shej043401.ww1.pdf]

Prospective external validation of the Predicting Out-of-OFfice Blood Pressure (PROOF-BP) strategy for triaging ambulatory monitoring in the diagnosis and management of hypertension: an observational cohort study

Supplemental Material

James P Sheppard, *Senior Research Fellow,*[1] Una Martin, *Professor,*[2] Paramjit Gill, *Professor,*[3] Richard Stevens, *Associate Professor,*[1] Richard Hobbs, *Professor,*[1] Jonathan Mant, *Professor,*[4] Marshall Godwin, *Professor,*[5] Janet Hanley, *Reader,*[6] Brian McKinstry, *Professor,*[7] Martin Myers, *Professor,*[8] David Nunan, *Senior Research Fellow*[1] Richard J McManus, *Professor*[1] on behalf of the PROOF-BP investigators

[1]Nuffield Department of Primary Care Health Sciences, University of Oxford, Oxford, UK
[2]Institute of Clinical Sciences, University of Birmingham, Birmingham, UK
[3]Warwick Medical School, University of Warwick, Coventry, UK
[4]University of Cambridge, Cambridge, UK
[5]Memorial University of Newfoundland, St John's, Canada
[6]Edinburgh Napier University, Edinburgh, UK
[7]University of Edinburgh, Edinburgh, UK
[8]University of Toronto, Toronto, Canada

Contents

1. **eFigure 1.** Definitions of normotension, sustained hypertension, white coat hypertension and masked hypertension
2. **eFigure 2.** PROOF-BP algorithm
3. **eFigure 3.** PROOF-BP strategy
4. **Extended methods**
5. **eTable 1.** Blood pressure monitor details
6. **eTable 2.** Strategy for blood pressure measurement according to guidelines
7. **eTable 3.** Sample size estimates and the impact on the precision of the study results, taking into account uncertainty about the expected prevalence of normotension and white coat hypertension
8. **eTable 4.** Blood pressure monitoring information
9. **eTable 5.** Accuracy of the PROOF-BP strategy compared to other strategies for the diagnosis of hypertension
10. **eTable 6.** Performance of the PROOF-BP strategy compared to other strategies for the diagnosis of hypertension
11. **eTable 7.** Sensitivity analyses
12. **eTable 8.** Post-hoc comparisons of the PROOF-BP strategy and guideline recommended approaches without the use of ABPM.
13. **Statistical analysis plan**
14. **References**

eFigure 1. Definitions of normotension, sustained hypertension, white coat hypertension and masked hypertension

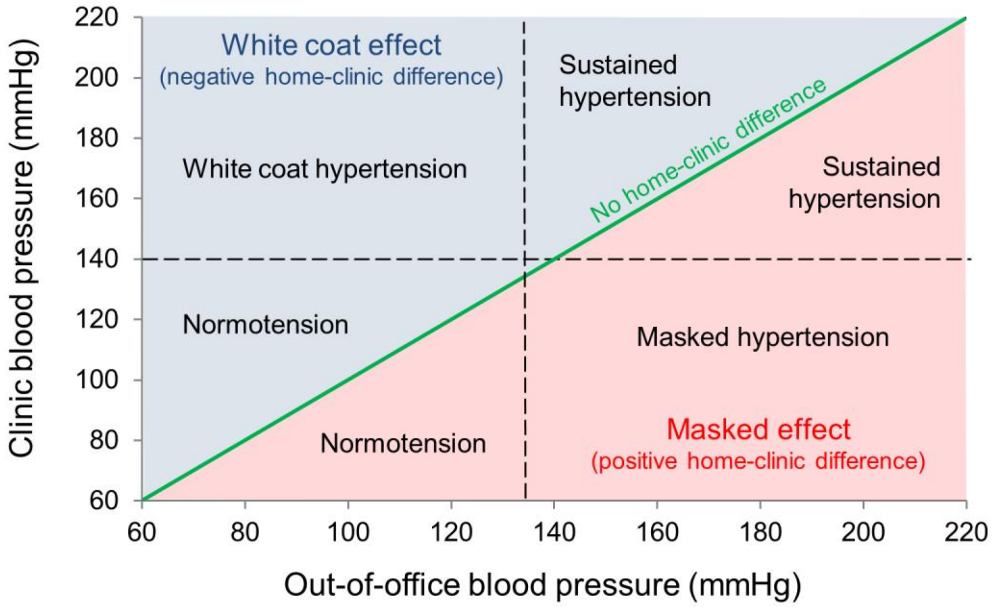

Condition	Clinic blood pressure	Out-of-office blood pressure	Home-clinic blood pressure difference
Normotension	<140/90mmHg	<135/85mmHg	Positive or negative
Sustained hypertension	≥140/90mmHg	≥135/85mmHg	Positive or negative
White coat hypertension	≥140/90mmHg	<135/85mmHg	Negative
Masked hypertension	<140/90mmHg	≥135/85mmHg	Positive
White coat effect	> out-of-office BP	< clinic BP	Negative
Masked effect	< out-of-office BP	> clinic BP	Positive

eFigure 2. PROOF-BP algorithm

$\textbf{out of office sBP} = clinic\ sBP + \big(33.57419 + (0.6269306\ \times age) + (-3.598565\ \times male\ sex) + (-0.036267\ \times clinic\ sBP) + (0.3617946\ \times sBP\ change) + (-0.2093273\ \times BMI) + (-5.069816\ \times diagnosis\ of\ hypertension) + (0.175593\ \times duration\ of\ hypertension) + (6.942526\ \times antihypertensive\ prescription) + (-0.6181946\ \times pulse\ pressure) + (-0.0077222\ \times [age \times clinic\ sBP]) + (0.009603\ \times [age \times pulse\ pressure]) + (0.2976424\ \times [sex \times BMI]) + (-0.2587568\ \times [sex \times duration\ of\ hypertension]) + (-14.73537 \times [sex \times antihypertensive\ prescription]) + (13.3899\ \times [sex \times diagnosis\ of\ hypertension])\big)$

$\textbf{out of office dBP} = clinic\ dBP + \ (59.34239 + (-0.3340627\ \times age) + (3.328766\ \times male\ sex) + (-0.4658008\ \times clinic\ dBP) + (-0.4040305\ \times dBP\ change) + (-0.6620654 \times BMI) + (-0.0296759\ \times diagnosis\ of\ hypertension) + (10.45892\ \times antihypertensive\ prescription) + (-11.07324\ \times diagnosis\ of\ cardiovascular\ disease) + \ (-0.060376\ \times pulse\ pressure) + (0.0118679\ \times [age \times dBP\ change]) + (0.0097736\ \times [age \times BMI]) + (0.1766609\ \times [age \times diagnosis\ of\ cardiovascular\ disease]) + (-0.133922\ \times [age \times antihypertensive\ prescription]) + (-7.997599\ \times [sex \times antihypertensive\ prescription]) + (4.628107\ \times [sex \times diagnosis\ of\ hypertension]))$

sBP=systolic blood pressure; dBP=diastolic blood pressure; BMI=body mass index
Binary variables coded as yes (1) or no (0)

eFigure 3.
PROOF-BP strategy

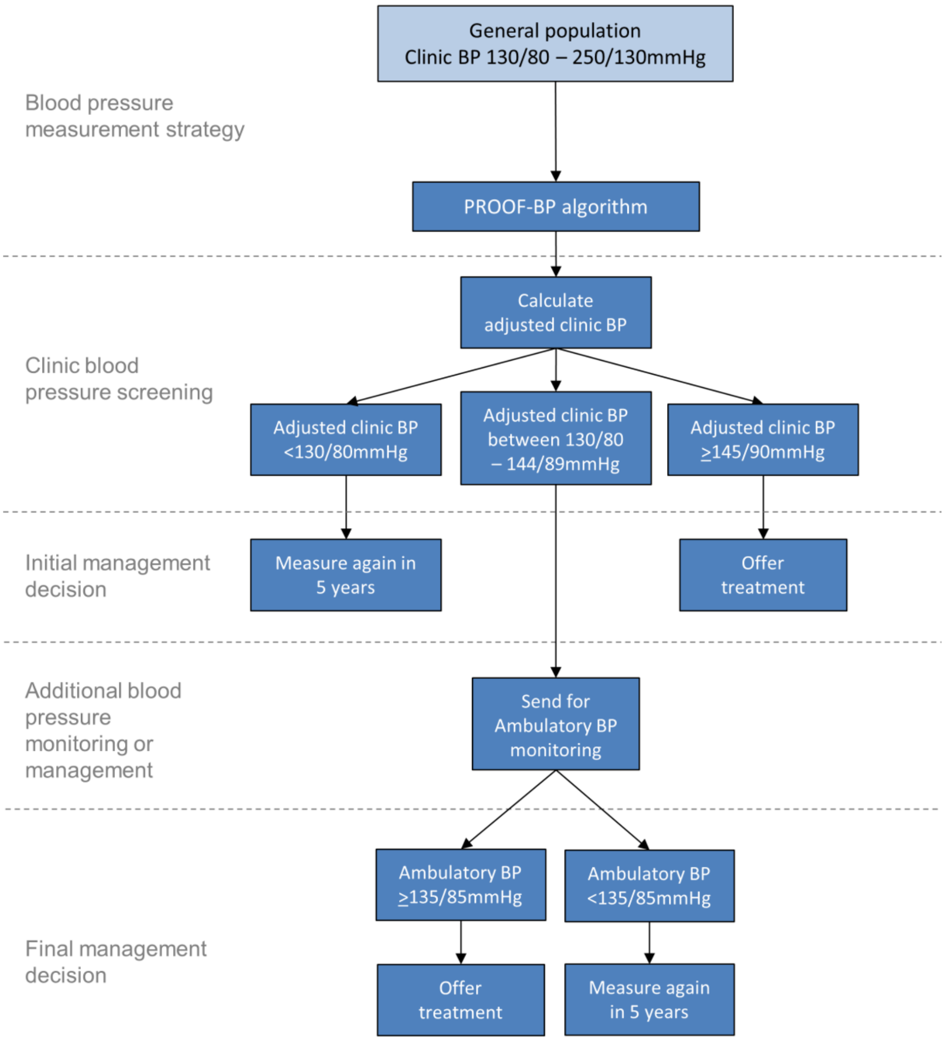

Extended methods

The protocol for this study have been published previously.[1]

Study design
This study used a prospective, multi-centre observational cohort design, recruiting patients from Primary Care and Secondary Care.

Study participants and setting
Consecutive patients attending participating centres in Primary or Secondary Care, for whom ambulatory blood pressure monitoring was considered appropriate, were enrolled between May 2015 and January 2017. Eligible patients were those undergoing ambulatory blood pressure monitoring (ABPM) as a result of routine blood pressure screening or monitoring in Primary Care or via referral to Secondary Care with suspected hypertension, newly diagnosed or treated hypertension, resistant hypertension, secondary hypertension or other conditions requiring specialist advice. Anonymised data were collected on all patients fulfilling the eligibility criteria:

Inclusion Criteria

- Age 18 years or above
- Attending clinical practice for routine ABPM

Exclusion Criteria

- Aged under 18 years old
- Lack of availability of basic clinical information
- Multiple clinic blood pressure readings (obtained on at least three occasions within the same visit) not recorded
- Ambulatory blood pressure monitor not worn as instructed

Procedures
All participants underwent ABPM, clinic blood pressure measurement and collection of patient characteristics. Data collected for each participant are detailed in the protocol[1] and included: blood pressure measurements (values and measurement technique), previous treatment prescriptions, body mass index (BMI), smoking status and history of diabetes, chronic kidney disease, atrial fibrillation and cardiovascular disease. Antihypertensive medication changes made after ABPM were not recorded. All data were collected from electronic health records and entered directly onto the study database by trained staff at each data collection site.

To capture as close to 'routine' blood pressures as possible, all participating sites were asked to measure clinic and ambulatory blood pressure according to their usual practice. No specific protocol for measurement was used. 'Routine blood pressure' was defined as readings taken by the consulting healthcare professional as part of routine clinical practice.

A minimum of three clinic readings taken at the time of referral for ambulatory blood pressure monitoring or at monitor fitting were required for inclusion in the study. It is acknowledged that this may not always reflect routine practice[2] (even though it is recommended in guidelines),[3] but this was

necessary to permit validation of the triaging algorithm. Each site was offered a validated automated blood pressure monitoring device (Omron M10-IT, Omron Corporation, Kyoto, Japan) to assist with the collection of multiple clinic blood pressure readings, but were given the option to continue using their own monitor, so long as at least three readings were taken and recorded. To our knowledge, all readings were taken with the physician or nurse present in the room. ABPM was conducted using the practice/hospital's own ambulatory monitor and fitted by a trained nurse or allied health professional. Some practices in Primary Care only collected daytime ambulatory pressures. Details of clinic and ambulatory blood pressure monitors used at each site are given in the online appendix (eTable 1, online appendix).

For the primary analysis, clinic blood pressure was defined by the 1st clinic reading taken at each visit, replicating the definitions used in the original PROOF-BP analyses[4] and better reflecting how blood pressure is captured in routine practice.[2,5] Mean of daytime ambulatory blood pressure was defined as it was used for routine practice – i.e. by patient diary, and/or monitor settings pre-programmed by the treating physician or nurse.

The PROOF-BP triaging approach

The triaging strategy applies an algorithm to clinic blood pressure readings and patient characteristics to identify three groups: those with definitively normal blood pressure, those with definitively high blood pressure and those requiring further investigation using ABPM. The algorithm utilises three blood pressures taken at the clinic appointment combined with information from the patient's electronic health record: age, sex, BMI, hypertensive and treatment history and the presence of cardiovascular disease (see eFigures 2 and 3, online appendix).[4] The triaging strategy is applied in three stages:[4]

1) Using the algorithm incorporating three clinic BP readings and patient characteristics, estimate the predicted difference between clinic and daytime ambulatory blood pressure.
2) Add this difference to the clinic blood pressure to generate an 'adjusted clinic blood pressure'.
3) Triage an individual for ambulatory monitoring (or not) depending on the level of adjusted clinic blood pressure (<130/80 mmHg = normal blood pressure not requiring treatment; 130/80-144/89mmHg = uncertain blood pressure requiring additional monitoring with ABPM; >145/90 mmHg = high blood pressure requiring treatment).

Primary outcome

The primary outcome of this study was the proportion correctly classified with hypertension using the triaging strategy compared to the reference standard of daytime ABPM (using a threshold for hypertension of ≥135/85 mmHg).[3,6,7] This was defined as the proportion of patients with sustained hypertension (true positives), normotension (true negatives), white coat hypertension (false positives) and masked hypertension (false negatives).

Secondary outcomes

The sensitivity (for detecting hypertension in patients with the condition), specificity (for ruling out hypertension in those without the condition), positive and negative predictor values were estimated and compared to guideline strategies for measuring blood pressure from the UK,[3] US,[7] Europe,[6] Canada[8] and Japan[9] (eTable 2, online appendix). Further secondary outcomes included accuracy of

the triaging strategy in different sub-groups: setting (Primary vs. Secondary Care), age (<65 years vs. ≥65 years), sex, smoking status (never/ex-smoker vs. current smoker), BMI (<30kg/m^2 vs. ≥30kg/m^2), previous history of hypertension, diabetes, chronic kidney disease and cardiovascular disease.

Data analysis

Descriptive statistics were used to describe the number of patients classified with sustained hypertension, white coat hypertension, normotension and masked hypertension with the triaging approach (the primary outcome) using daytime ABPM as the reference standard. These were used to calculate the sensitivity, specificity, positive and negative predictor values of the triaging approach and the total proportion of participants with correctly classified hypertensive status and proportion that would have been referred for ABPM.

To examine model performance, a logistic regression model was constructed with true hypertension (defined by daytime ABPM) as the dependant outcome variable and classification using the triaging approach as the independent predictor variable. From this model the area under the receiver operating characteristic (AUROC) curve statistic was estimated. For secondary analyses, classification of patients' hypertensive status and utilisation of ABPM with the PROOF-BP strategy was compared to guideline recommended strategies for the diagnosis of hypertension[3,6-9] (eTable 2) with McNemar's chi-squared test.

Further analyses were conducted examining the primary outcomes using different definitions of clinic and ambulatory blood pressure:

1) Mean of the 2nd & 3rd clinic readings
2) Mean of all three clinic readings
3) Mean daytime ambulatory blood pressure defined as 'up-to-standard' (estimated from a minimum of 14 readings)[3]
4) Mean daytime ambulatory blood pressure defined as 'up-to-standard', with outlying readings excluded[10] and standardised times for defining the daytime period (7am-11pm; derived from raw ambulatory blood pressure data)
5) Mean 24 hr ambulatory blood pressure (all available readings)
6) Mean 24 hr ambulatory blood pressure defined as 'up-to-standard' (estimated from a minimum of 14 daytime readings and 7 night-time readings [if readings are taken at 30 minute intervals] or 70% of attempted night-time readings)[3]
7) Mean night-time ambulatory blood pressure (all available readings)
8) Mean night-time ambulatory blood pressure defined as up-to-standard readings (estimated from a minimum of 7 readings [if readings are taken at 30 minute intervals] or 70% of attempted readings).
9) Mean night-time ambulatory blood pressure defined as 'up-to-standard', with outlying readings excluded[10] and standardised times for defining the daytime period (11pm-7am; derived from raw ambulatory blood pressure data)

Post-hoc analyses

Post-hoc analyses were undertaken to examine performance of the PROOF-BP algorithm on its own (without additional ambulatory monitoring) and this was compared to other blood pressure measurement strategies (employed without ABPM).[10 11 30-32] Subgroup analyses of the sensitivity and

specificity of the PROOF-BP triaging approach were undertaken and accompanied by one additional non pre-specified subgroup: patients in whom the treating clinician's own monitor was used to measure clinic blood pressure vs. those where monitors were provided by the research team.

All analyses were conducted using STATA version 13.1 (MP parallel edition, StataCorp, Texas, USA). Results are presented as means or proportions, with standard deviations or 95% confidence intervals, unless otherwise stated.

Sample size
Based on the original validation of the PROOF-BP prediction model,[4] accrual of data from at least 800 patients was required for estimation of hypertensive status with an accuracy of ±1-3% (see eTable 3, online appendix).[1] A sample size of up to 1000 patients was specified to ensure that the pre-specified sub-group analysis could be adequately powered. Approximately 364 patients (182 in each group) were required in each sub-group to examine the secondary outcomes proposed in the proposed study. This was based on a likelihood ratio test of two proportions detecting a 10% difference in the classification of hypertensive status between two sub-group populations, with a significance level of 0.05 and 90% power.

Patient and public involvement
Patients with a history of hypertension were approached to discuss the study at the design phase of the project. In particular their opinions were sought on the methods of recruitment and patient facing study literature, prior to ethics and NHS R&D applications.

Approvals
Ethical approval for this study was obtained from the National Research Ethics Service Committee South Central – Oxford A (reference; 15/SC/0184), and site-specific R&D approval acquired from the relevant NHS trusts.

eTable 1. Blood pressure monitor details

Site	Clinic BP monitor used	ABPM monitor used
GP surgery 1	Omron M10-IT	A&D TM-2430
GP surgery 2	Omron M7 (HEM-780-E)	SpaceLabs 90217
GP surgery 3	Omron HEM-907-E	A&D TM-2430/Suntech Oscar 2
GP surgery 4	Omron M10-IT	Watch BP 03
GP surgery 5	Omron M10-IT	Watch BP 03
GP surgery 6	Omron M10-IT	Welch Allyn 6100 Series
GP surgery 7	Omron M10-IT	SpaceLabs 90217
GP surgery 8	Omron M10-IT	n/a
GP surgery 9	n/a	n/a
GP surgery 10	Omron M10-IT	SpaceLabs 90217
Hospital site	Microlife Watch BP Office	SpaceLabs 90217

n/a = not available (missing data) due to multiple devices being used and/or device name and model used during data collection not being documented.

eTable 2. Strategy for blood pressure measurement according to guidelines

Guideline	Year	Clinic blood pressure definition	ABPM	Threshold for hypertension
National Institute for health Clinical Excellence (NICE)[3]*	2011	Lowest of the 1 & 2nd or 3rd if the initial pair of readings are both raised	Daytime	>180/110mmHg (clinic) or >135/85mmHg (ABPM)
European Society for Hypertension (ESH)[6]	2013	Mean of the 2nd & 3rd readings	Not routinely recommended	>140/90mmHg
Japanese Society of Hypertension (JSH)[9]	2014	Mean of the 1st & 2nd readings or mean of 3 if the initial pair of readings are >5mmHg apart	Not routinely recommended	>140/90mmHg
Canadian Hypertension Education Programme (CHEP)[8]	2015	Mean of 2nd to the 6th readings taken using an automated blood pressure monitor	Not routinely recommended	>135/85mmHg
American College of Cardiology/American Heart Association Task Force[7]*	2015	Mean of the 1st & 2nd readings or mean of 3 if the initial pair of readings are >5mmHg apart	Daytime	>135/85mmHg (ABPM)
PRedicting Out-of-OFfice Blood Pressure in the clinic algorithm (PROOF-BP)[4]†	2016	Adjusted clinic blood pressure using the PROOF-BP algorithm	Daytime	>145/90mmHg (adjusted clinic) or >135/85mmHg (ABPM)

ABPM = ambulatory blood pressure monitoring

*Patients with raised clinic BP (>140/90 mmHg) should be referred for out-of-office monitoring (diagnosis only)

†Patients with an adjusted clinic BP between >130/80 and 144/89 mmHg should be referred for out-of-office monitoring

eTable 3. Sample size estimates and the impact on the precision of the study results, taking into account uncertainty about the expected prevalence of normotension and white coat hypertension

**Scenario**	**Sample size**	**Sustained hypertensive (true positive)**		**Normotensive (true negative)**		**White coat hypertensive (false positive)**		**Masked hypertensive (false negative)**	
		**Point estimate**	**Predicted CI**	**Point estimate**	**Predicted CI**	**Point estimate**	**Predicted CI**	**Point estimate**	**Predicted CI**
Prevalences observed in the original PROOF-BP study[4]	1000	71%	68-74%	24%	21-27%	3%	2-4%	2%	1-3%
	800	71%	68-74%	24%	21-27%	3%	2-4%	2%	1-3%
Prevalence of normotension decreases by 50%	1000	83.5%	81-86%	12%	10-14%	3.5%	2-5%	1%	0.5-2%
	800	83.5%	81-86%	12%	10-14%	3.5%	2-5%	1%	0.5-2%
Prevalence of normotension decreases by 75%	1000	89.5%	87-91%	6%	5-8%	4%	3-5%	0.5%	0-1%
	800	89.5%	87-92%	6%	4-8%	4%	3-6%	0.5%	0-1%
Prevalence of white coat hypertension increases by 50%	1000	69.5%	67-72%	24%	21-27%	4.5%	3-6%	2%	1-3%
	800	69.5%	66-73%	24%	21-27%	4.5%	3-6%	2%	1-3%
Prevalence of white coat hypertension increases by 100%	1000	68%	65-71%	24%	21-27%	6%	5-8%	2%	1-3%
	800	68%	65-71%	24%	21-27%	6%	4-8%	2%	1-3%

CI=confidence interval; PROOF-BP=predicting out-of-office blood pressure in the clinic

eTable 4. Blood pressure monitoring information

Characteristic	Primary Care	Secondary Care
**Clinic blood pressure measurement:**		
- Left arm used	205 (58.7%)	530 (99.4%)
- Right arm used	144 (41.3%)	3 (0.6%)
- Unknown	5 (1.4%)	0 (0%)
Median number of readings		
- Systolic	3	6
- Diastolic	3	6
**Ambulatory blood pressure measurement**:		
- Left arm used	273 (77.1%)	436 (81.8%)
- Right arm used	66 (18.6%)	91 (17.1%)
- Unknown	15 (4.2%)	6 (1.1%)
Median number of readings		
- Daytime	22	28
- Night time	7	9
- 24hr	30	37
Reason for referral		
Suspected hypertension/uncontrolled hypertension	263 (73.9%)	257 ((47.5%)
Specialist treatment advice	2 (0.6%)	89 (16.5%)
Young hypertensive	0 (0.0%)	49 (9.1%)
Suspected white coat hypertension	31 (8.7%)	14 (2.6%)
Secondary hypertension	0 (0.0%)	28 (5.2%)
Suspected resistant hypertension	0 (0.0%)	23 (4.3%)
Side effects to treatment	1 (0.3%)	16 (3.0%)
Hospital admission for related condition	2 (0.6%)	12 (2.2%)
Severe hypertension	0 (0.0%)	10 (1.8%)
Medication change	4 (1.1%)	4 (0.7%)
Accelerated hypertension	0 (0.0%)	5 (0.9%)
Suspected hypotension	3 (0.8%)	1 (0.2%)
Other	50 (14.0%)	33 (6.1%)

eTable 5. Accuracy of the PROOF-BP strategy compared to other strategies for the diagnosis of hypertension

Diagnostic strategy	True positive (Sustained hypertensive)		True negative (normotensive)		False positive (white coat hypertensive)		False negative (masked hypertensive)		Correctly classified		Utilisation of ABPM	
	**No.**	**(%)**	**No.**	**(%)**	**No.**	**(%)**	**No.**	**(%)**	**No.**	**(%)**	**No.**	**(%)**
PROOF-BP	584	65.8%	217	24.5%	69	7.8%	17	1.9%	801	90.3%	435	49.0%
NICE	467	52.6%	284	32.0%	2	0.2%	134	15.1%	751	84.7%*	541	61.0%*
USPSTF	518	58.4%	286	32.2%	0	0.0%	83	9.4%	804	90.6%	687	77.5%*
ESH	494	55.7%	128	14.4%	158	17.8%	107	12.1%	622	70.1%*	0	0.0%*
JSH	506	57.0%	122	13.8%	164	18.5%	95	10.7%	628	70.8%*	0	0.0%*
CHEP†	549	61.9%	88	9.9%	198	22.3%	52	5.9%	637	71.8%*	0	0.0%*

*Significantly different from PROOF-BP approach (comparison using McNemar's chi-squared test; $p<0.001$)
†Clinic BP in patients from Primary Care estimated from 3 readings

eTable 6. Performance of the PROOF-BP strategy compared to other strategies for the diagnosis of hypertension

Diagnostic strategy	**AUROC** (95% CI)		Sensitivity	Specificity	Positive predictive value	Negative predictive value
PROOF-BP	0.86	0.84 - 0.89	97.2%	75.9%	89.4%	92.7%
NICE	0.89	0.87 - 0.90	77.7%	99.3%	99.6%	67.9%
USPSTF	0.93	0.92 - 0.94	86.2%	100.0%	100.0%	77.5%
ESH	0.63	0.60 - 0.67	82.2%	44.8%	75.8%	54.5%
JSH	0.63	0.60 - 0.67	84.2%	42.7%	75.5%	56.2%
CHEP†	0.61	0.58 - 0.64	91.4%	30.8%	73.5%	62.9%

AUROC=Area under the receiver operating characteristic curve
†Clinic BP in patients from Primary Care estimated from 3 readings

eTable 7. Sensitivity analyses

Blood pressure measurement	Definition	Total population	True positive (Sustained hypertensive)		True negative (normotensive)		False positive (white coat hypertensive)		False negative (masked hypertensive)		Correctly classified		Utilisation of ABPM	
			No.	(%)	No.	(%)	No.	(%)	No.	(%)	No.	(%)	No.	(%)
Clinic blood pressure	Mean of 2nd & 3rd readings	887	568	64.0%	231	26.0%	55	6.2%	33	3.7%	799	90.1%	452	51.0%
	Mean of 3 readings	887	575	64.8%	229	25.8%	57	6.4%	26	2.9%	804	90.6%	451	50.8%
Ambulatory blood pressure	Daytime (up to standard readings)	799	525	65.7%	203	25.4%	59	7.4%	12	1.5%	728	91.1%	385	48.2%
	Daytime (standardised time [7am-11pm])*	637	505	79.3%	92	14.4%	21	3.3%	19	3.0%	597	93.7%	297	46.6%
	24 hour (all readings)	850	598	70.4%	179	21.1%	52	6.1%	21	2.5%	777	91.4%	411	48.4%
	24 hour (up to standard readings)	709	497	70.1%	155	21.9%	45	6.3%	12	1.7%	652	92.0%	340	48.0%
	Nighttime (all readings)	781	463	59.3%	220	28.2%	85	10.9%	13	1.7%	683	87.5%	375	48.0%
	Nighttime (up to standard readings)	706	406	57.5%	211	29.9%	80	11.3%	9	1.3%	617	87.4%	340	48.2%
	Nighttime (standardised time [11pm-7am])*	546	313	57.3%	153	28.0%	71	13.0%	9	1.6%	466	85.3%	254	46.5%

*Raw data files used to calculate standardised time blood pressure estimates were not available for all patients

eTable 8. Post-hoc comparisons of the PROOF-BP strategy and guideline recommended approaches without the use of ABPM.

**Diagnostic strategy**	**AUROC** (95% CI)		**Sensitivity**	**Specificity**	**Positive predictive value**	**Negative predictive value**
PROOF-BP	0.66	0.63 - 0.69	67.4%	64.7%	80.0%	48.5%
NICE	0.66	0.62 - 0.69	77.7%	54.6%	78.2%	53.8%
USPSTF	0.64	0.60 - 0.67	86.2%	40.9%	75.4%	58.5%
ESH	0.63	0.60 - 0.67	82.2%	44.8%	75.8%	54.5%
JSH	0.63	0.60 - 0.67	84.2%	42.7%	75.5%	56.2%
CHEP†	0.61	0.58 - 0.64	91.4%	30.8%	73.5%	62.9%

AUROC=Area under the receiver operating characteristic curve

†Clinic BP in patients from Primary Care estimated from 3 readings

PROOF-ABPM

Statistical analysis plan

Study Title: Prospective Register Of patients undergoing repeated OFfice and Ambulatory Blood Pressure Monitoring (PROOF-ABPM)

Statistical Analysis Plan

Version number and date: V1.0 06.04.17

Principal Investigator: **Dr James Sheppard**, Nuffield Department of Primary Care Health Sciences, University of Oxford, Oxford.

Investigators:
Professor Richard McManus, Nuffield Department of Primary Care Health Sciences, University of Oxford, Oxford.
Professor Una Martin, School of Clinical and Experimental Medicine, University of Birmingham, Birmingham.
Professor Paramjit Gill, Primary Care Clinical Sciences, University of Birmingham, Birmingham.
Professor Richard Stevens, Nuffield Department of Primary Care Health Sciences, University of Oxford, Oxford.
Mr David Yeomans, Lay patient advisor.

Version History

Version:	Version Date:	Changes:

TABLE OF CONTENTS

Table of Contents 17
1 Study design 18
1.1 Outcomes measures 18
1.1.1 Primary outcome 18
1.1.2 Secondary outcomes 19
1.2 Target population 19
1.2.1 Inclusion Criteria 19
1.2.2 Exclusion Criteria 19
1.3 Sample size 20
2 Analysis – General considerations 21
2.1 Descriptive statistics 21
2.2 Definition of population for analysis 21
2.3 Definition of variables for analysis 21
3 PRIMARY ANALYSIS 22
3.1 Primary outcome 22
3.2 Handling missing data 22
3.3 Handling outliers 23
4 SECONDARY ANALYSIS 23
5 SENSITIVITY ANALYSIS 23
6 SUBGROUP ANALYSES 24
7 ADDITIONAL EXPLORATORY ANALYSIS 24
8 CHANGES TO THE PROTOCOL OR PREVIOUS VERSIONS OF SAP 24
9 References 10
10 Appendices .... **Error! Bookmark not defined.**
10.1 Appendix I. Data tables/figures for analysis write up .... **Error! Bookmark not defined.**

1 STUDY DESIGN

The aim of this study is to examine novel strategies for the diagnosis and management of hypertension using data from routine clinical practice. This will be achieved using a prospective, multi-centre observational cohort study design, setting up a Prospective Register Of patients undergoing repeated OFfice and Ambulatory Blood Pressure Monitoring (PROOF-ABPM) in Primary Care, Secondary Care and at pharmacies. Data contained within the register will include patient characteristics, repeated clinic and ambulatory blood pressure, clinical assessment data and subsequent admissions to hospital and mortality. The PROOF-ABPM will be unique in its consideration of multiple clinic blood pressure measurements in relation to ambulatory blood pressure readings taken in routine clinical practice.

1.1 OUTCOMES MEASURES

Priority	Objectives	Outcome Measures
**Primary objective**	Establish the accuracy of the PROOF-BP prediction tool at predicting out-of-office blood pressure in routine clinical practice	The proportion of true positive, true negative, false positive and false negative classifications of hypertension according to out-of-office monitoring.
**Secondary objectives**	Establish the accuracy of the PROOF-BP prediction tool compared to current strategies in routine clinical practice	Improvement in the classification of patients' hypertensive status of >10% or reduction in the utilisation of out-of-office monitoring of >20% compared to existing strategies.
	Establish the accuracy of the PROOF-BP prediction tool in different clinical settings (Primary Care, Secondary Care and pharmacies)	Difference in the proportion of patients correctly classified as hypertensive (according to out-of-office monitoring) by setting.
	Establish the accuracy of the PROOF-BP prediction tool in specific populations (older vs. younger patients, males vs. females, those at high cardiovascular disease risk vs. low risk, those taking antihypertensive medications vs. those not)	Difference in the proportion of patients correctly classified as hypertensive (according to out-of-office monitoring) by patient characteristic.
**Exploratory objectives**	Examine whether the 'adjusted clinic blood pressure' generated by the prediction model predicts long term clinical outcomes (e.g. hospital admission with myocardial infarction or stroke, mortality) better than standard clinic blood pressure	Hazard ratio describing the association between adjusted clinic blood pressure and total mortality, cardiovascular mortality, hospital admission with stroke, myocardial infarction or heart failure.

1.1.1 PRIMARY OUTCOME

The primary outcome of this study will be to define the accuracy of the PROOF-BP prediction tool in terms of the proportion of true/false positive/negative results in the general population attending routine clinical practice.

1.1.2 Secondary outcomes

Secondary outcomes will include assessment of model accuracy in different sub-groups – age (young vs. old), cardiovascular disease risk (high vs. low risk according to previous history and risk scores using data where available), those with Chronic Kidney Disease, Diabetes and across healthcare settings: Primary Care, Secondary Care and pharmacy settings.

In the longer term, linked data from the registry will be used to examine whether the 'adjusted clinic blood pressure' generated by the prediction model can better predict long term clinical outcomes (e.g. hospital admission with myocardial infarction/stroke and mortality) than standard clinic blood pressure. After the initial period of data collection is complete, the resources and funding required to continue ongoing data collection and follow-up will be reviewed. Where possible, patient accrual and data collection will continue and the study will become a research database permitting further investigations into blood pressure monitoring by a variety of means and cardiovascular disease risk factor data linked to cardiovascular disease morbidity and mortality in routine clinical practice.

1.2 Target population

The register is web-based to permit access from a variety of healthcare settings. Data collection procedures will be piloted in the hypertension clinic at University Hospitals Birmingham. Following successful role out in this clinic, other centres from Primary care will be invited to contribute patient data to the registry. Eligible patients will meet the following inclusion/exclusion criteria:

1.2.1 Inclusion Criteria

- Male and female subjects
- Age ≥18 years
- Attending routine clinical practice for ambulatory blood pressure monitoring

1.2.2 Exclusion Criteria

The participant may not enter the study if ANY of the following apply:

- Lack of availability of basic clinical information
- Clinic blood pressure readings obtained on at least three occasions within the same visit not recorded
- Ambulatory blood pressure monitor not worn as instructed and/or invalid readings

1.3 SAMPLE SIZE

The proposed study will collect data on consecutive patients referred for ambulatory blood pressure monitoring in routine clinical practice. Based on the initial validation phase of the PROOF-BP prediction model, conducted using data from previous studies,[4] accrual of data from approximately 1000 patients would allow for estimation of hypertensive status with an accuracy of ±1-3%. In this previous data, 71% of patients were classed as true positives, 24% were classed as true negatives, 3% were classed as false positives and 2% were classed as false negatives. In population of 1000 patients it would be possible to estimate these rates with the following 95% confidence intervals: true positive 71% (68-74%), true negative 24% (21-27%), false positive 3% (2-4%) and false negative 2% (1-3%).

Table 1. Sample size estimates and the impact on the precision of the study results

**Scenario**	**Sample size**	**Sustained hypertensive (true positive)**		**Normotensive (true negative)**		**White coat hypertensive (false positive)**		**Masked hypertensive (false negative)**	
		**Point estimate**	**Predicted CI**	**Point estimate**	**Predicted CI**	**Point estimate**	**Predicted CI**	**Point estimate**	**Predicted CI**
Prevalences observed in the original PROOF-BP study[4]	1000	71%	68-74%	24%	21-27%	3%	2-4%	2%	1-3%
	800	71%	68-74%	24%	21-27%	3%	2-4%	2%	1-3%
Prevalence of normotension decreases by 50%	1000	83.5%	81-86%	12%	10-14%	3.5%	2-5%	1%	0.5-2%
	800	83.5%	81-86%	12%	10-14%	3.5%	2-5%	1%	0.5-2%
Prevalence of normotension decreases by 75%	1000	89.5%	87-91%	6%	5-8%	4%	3-5%	0.5%	0-1%
	800	89.5%	87-92%	6%	4-8%	4%	3-6%	0.5%	0-1%
Prevalence of white coat hypertension increases by 50%	1000	69.5%	67-72%	24%	21-27%	4.5%	3-6%	2%	1-3%
	800	69.5%	66-73%	24%	21-27%	4.5%	3-6%	2%	1-3%
Prevalence of white coat hypertension increases by 100%	1000	68%	65-71%	24%	21-27%	6%	5-8%	2%	1-3%
	800	68%	65-71%	24%	21-27%	6%	4-8%	2%	1-3%

CI=confidence interval; PROOF-BP=predicting out-of-office blood pressure in the clinic

Approximately 182 patients would be required in each sub-group to examine the secondary outcomes proposed in the proposed study. This is based on a likelihood ratio test of two proportions detecting a 10% difference in the classification of hypertensive status between two sub-group populations (Secondary care vs. Primary care, Primary care vs. pharmacies, older vs. younger patients, high vs. low risk patients, etc.) with a significance level of 0.05 and power of 0.9. Assuming correct classification of 95% of patients in one group and 85% in the other, approximately 364 patients (182 in each group)

would be required to demonstrate a significant difference. Thus, our recruitment target of 1000 patients should be sufficient to answer the secondary outcomes provided recruitment is appropriately distributed across clinic settings and patient characteristic sub-groups.

2 Analysis – General considerations

2.1 Descriptive statistics

Descriptive statistics will be used to define the study population at baseline, given from the total population, and separately by those recruited from primary and secondary care. Patient characteristics will include demographic data on age, gender, ethnic origin and other baseline characteristics including medical history (past and present), current therapies, and mean clinic and daytime ambulatory blood pressure levels. Summary information regarding the clinic and ambulatory blood pressure monitoring conducted will be also be present, including arm used for monitoring, number of readings taken and reason for referral for ambulatory monitoring. A full list of variables to be presented is listed in the blank data tables/figures given in appendix 1. All data will be presented as means ± standard deviation or proportions of the total population, unless otherwise stated.

2.2 Definition of population for analysis

The population eligible for both primary and secondary analyses will be all those attending routine clinical practice for ambulatory blood pressure monitoring in both primary and secondary care. Patients will be excluded from the analysis if they do not have data relating to clinic (three readings from the same visit) or mean daytime ambulatory blood pressure, are aged <18 years, or lack the basic clinical information required to calculated an adjusted clinic blood pressure using the PROOF-BP prediction tool (age, sex, BMI, history of hypertension, cardiovascular disease and/or antihypertensive treatment). A comparison of characteristics from included and excluded patients will be given as an additional table in the online appendix.

2.3 Definition of variables for analysis

Clinic blood pressure will presented as the mean of the 2nd and 3rd readings in the baseline characteristics. For the primary analysis, clinic blood pressure will be defined as 1st clinic reading taken, prior to fitting of the ambulatory blood pressure monitor, replicating the definitions used in the original PROOF-BP analyses.

Out-of-office blood pressure will be defined as the mean of all daytime ambulatory blood pressure readings recorded during ABPM. The daytime period will be defined as it was used for routine practice – *i.e.* defined by patient diary, and/or monitor settings pre-programmed by the treating clinician. Sensitivity analyses will explore the impact of limiting analyses to 'up-to-standard' ABPM readings. A minimum of 14 readings will be required to define an 'up-to-standard' daytime blood pressure.[3] Up-to-standard nighttime blood pressures will be defined as a minimum of 7 readings (if nighttime blood pressure is measured at 30 minute intervals) or 70% of attempted readings being present.[11] Mean 24hr blood pressure will be defined as all readings taken during the ABPM period.

All other variables will be defined as recorded from the participants' medical records. Sensitivity analyses will be conducted examining the primary outcomes using different definitions of clinic and ambulatory blood pressure (see section 5 below).

3 PRIMARY ANALYSIS

3.1 PRIMARY OUTCOME

The primary outcome of this study will be to define the accuracy of the PROOF-BP prediction tool in terms of the proportion of true/false positive/negative results in the general population attending routine clinical practice. The PROOF-BP algorithm will be used to estimate an adjusted clinic blood pressure which will then be compared to the corresponding ambulatory blood pressure (the reference standard) to identify the proportion of patients classified as true positives (sustained hypertensives), false positives (white coat hypertensives), true negatives (normotensives) and false negatives (masked hypertensives). Thresholds of blood pressure level to define hypertension by each measurement method are defined in table 2 (below). Where an adjusted clinic blood pressure would result in referral for ABPM (i.e. a blood pressure estimate of 130/80-145/90mmHg), hypertensive classification will be defined as correct (true positive/negative). Adjusted clinic and ambulatory blood pressures which are discordant from one another will be classified as false positive (white coat hypertensives) or false negatives (masked hypertensive), depending of the direction of disagreement.

Table 2. Classification of hypertensive status by blood pressure level and measurement strategy

Blood pressure level	Clinic blood pressure	Adjusted clinic blood pressure	Ambulatory blood pressure (reference)
<130/80 mmHg	Normotensive	Normotensive	Normotensive
130/80-134/84 mmHg	Normotensive	Refer for ABPM*	Normotensive
135/85-139/89 mmHg	Normotensive	Refer for ABPM*	Hypertensive
140/90-144/90 mmHg	Hypertensive	Refer for ABPM*	Hypertensive
≥145/90 mmHg	Hypertensive	Hypertensive	Hypertensive

*Defined as true positive or true negative depending on outcome of ABPM (i.e. not discordant from ABPM)

A logistic regression model will be constructed with true hypertension (defined by daytime ambulatory blood pressure monitoring) as the dependant outcome variable and adjusted clinic blood pressure (and/or hypertensive classification according to adjusted clinic blood pressure) as the independent predictor variable. The adjusted clinic blood pressure is already adjusted for age, sex, blood pressure level and change, BMI and medical history so no further adjustments will be made to the model. From this model, the sensitivity and specificity of adjusted clinic blood pressure for true hypertension will be estimated (using the thresholds defined above) and the area under the receiver operating characteristic (AUROC) curve statistic will be reported. Performance statistics will be estimated based on the PROOF-BP predicted classification used in conjunction with ambulatory blood pressure monitoring. Where appropriate, the AUROC for adjusted clinic blood pressure will be presented as a graph of sensitivity vs. 1 – specificity (see blank figures in online appendix).

3.2 HANDLING MISSING DATA

A complete case analysis will be conducted assuming data completeness of >90% across all variables. Where this is not the case, missing binary variables required to estimate the adjusted clinic blood pressure will be recoded. Individuals with missing data on history of hypertension and cardiovascular disease will be assumed to have no history of disease. Likewise, missing data on prescriptions will be assumed to have no drug prescriptions. Individuals with missing data relating to sex will be excluded.

All other covariates (age, BMI) used to estimate the adjusted clinic blood pressure which has a completeness of <90% will be imputed using multiple imputation. The presence of missing blood pressure readings is a reason for exclusion from the study and so these will not be imputed. Due to sporadic recording and dubious accuracy (particularly in secondary care), it was not possible to record time since diagnosis of hypertension as part of the minimum study dataset and therefore all participants will be given the value of zero for this variable. This will have no impact on the accuracy of the model where it is used in patients prior to a diagnosis of hypertension (as is most common in routine clinical practice).

3.3 HANDLING OUTLIERS

Continuous data will be cleaned and outlying values excluded where appropriate. Blood pressure outliers will be defined a systolic blood pressure of <70mmHg or >260mmHg and a diastolic blood pressure of <40mmHg or >150mmHg as previously described.[10] Height values of <100cm or 250cm> and weight values of <40kg or 150kg> will be recoded to missing unless it is explicitly defined as being correct in the medical notes.

4 SECONDARY ANALYSIS

Secondary aims of this analysis will be to compare the accuracy of the PROOF-BP prediction tool to current strategies in routine clinical practice. Specifically, chi-squared statistics will be used to compare the classification of patients' hypertensive status (% correctly classified) using the PROOF-BP prediction model with existing strategies proposed by NICE,[3] AHA/USPSTF,[12,13] ESH,[6] JSH[8] and CHEP[9] for the diagnosis and management of hypertension. A clinically significant difference will be defined as an improvement in patient classification of >10% or reduction in the utilisation of out-of-office monitoring of >20%. The performance of each diagnosis strategy will be examined using AUROC statistics as described above (section 3.1).

5 SENSITIVITY ANALYSIS

Sensitivity analyses will be conducted examining the primary analyses using different definitions of clinic and ambulatory blood pressure. Specifically, the proportion of true/false positive/negative results, sensitivity and specificity and AUROC of adjusted clinic blood pressure will be estimated from data using the following alternative definitions of clinic blood pressure and ambulatory blood pressure:

1) Daytime ambulatory blood pressure defined according to 'up-to-standard' readings (see section 2.3 for definitions)
2) mean of the 2nd & 3rd clinic readings taken

3) mean of all three clinic readings taken
4) mean 24 hr ambulatory blood pressure (all readings)
5) mean 24 hr ambulatory blood pressure (up-to-standard readings)
6) mean nighttime ambulatory blood pressure (all readings)
7) mean nighttime ambulatory blood pressure (up-to-standard readings)

6 SUBGROUP ANALYSES

Subgroup analyses will be undertaken to establish the accuracy of the PROOF-BP prediction tool in specific populations and settings. The proportion of patients with a correct classification of their hypertensive status and the proportion of patients being referred for out-of-office monitoring will be compared in patients with the following characteristics:

- Recruited from Primary care vs. Secondary care
- Age <65 years vs. ≥65 years
- Males vs. females
- Smoking status (current vs. ex/never)
- BMI (± 30kg/m^2)
- Diagnosis of hypertension vs. no diagnosis
- Antihypertensive treatment vs. no treatment
- Diabetes vs. no diabetes
- CKD vs. no CKD
- History of CVD vs. no history of CVD

Subgroup analyses with at least 182 patients within each group will be sufficiently powered to detect a significant difference in hypertensive classification/ambulatory blood pressure monitoring utilisation.

7 ADDITIONAL EXPLORATORY ANALYSIS

For all consenting patients, study data will be linked to the Office for National Statistics (ONS) mortality register and Hospital Episodes Statistics (HES) database via NHS Digital's Data Linkage and Extract Service. This will allow patient events to be tracked following enrolment into the study. These linked data will be used to examine the association between the 'adjusted clinic blood pressure' (estimated from the PROOF-BP prediction model) and clinical outcomes such as death, cardiovascular disease, myocardial infarction and stroke. Such analyses will be conducted using Cox proportional hazards models after sufficient outcome data (and patients years of follow-up) have been accrued.

8 CHANGES TO THE PROTOCOL OR PREVIOUS VERSIONS OF SAP

No changes have been made.

References

1. Sheppard JP, Martin U, Gill P, Stevens R, McManus RJ. Prospective Register Of patients undergoing repeated OFfice and Ambulatory Blood Pressure Monitoring (PROOF-ABPM): protocol for an observational cohort study. *BMJ open* 2016; **6**(10): e012607.

2. Williams B. Time to Abandon Clinic Blood Pressure for the Diagnosis of Hypertension? *Circulation* 2016; **134**(23): 1808-11.

3. National Clinical Guideline Centre. Hypertension: clinical management of primary hypertension in adults; Clinical guideline 127. London: Royal College of Physicians (UK), 2011.

4. Sheppard JP, Stevens R, Gill P, et al. Predicting Out-of-Office Blood Pressure in the Clinic (PROOF-BP): Derivation and Validation of a Tool to Improve the Accuracy of Blood Pressure Measurement in Clinical Practice. *Hypertension* 2016; **67**(5): 941-50.

5. Martin U, Haque MS, Wood S, et al. Ethnicity and Differences Between Clinic and Ambulatory Blood Pressure Measurements. *American journal of hypertension* 2014.

6. Mancia G, Fagard R, Narkiewicz K, et al. 2013 ESH/ESC Guidelines for the management of arterial hypertension: the Task Force for the management of arterial hypertension of the European Society of Hypertension (ESH) and of the European Society of Cardiology (ESC). *Journal of hypertension* 2013; **31**(7): 1281-357.

7. Whelton PK, Carey RM, Aronow WS, et al. 2017 ACC/AHA/AAPA/ABC/ACPM/AGS/APhA/ASH/ASPC/NMA/PCNA Guideline for the Prevention, Detection, Evaluation, and Management of High Blood Pressure in Adults: A Report of the American College of Cardiology/American Heart Association Task Force on Clinical Practice Guidelines. *Journal of the American College of Cardiology* 2017.

8. Daskalopoulou SS, Rabi DM, Zarnke KB, et al. The 2015 Canadian Hypertension Education Program recommendations for blood pressure measurement, diagnosis, assessment of risk, prevention, and treatment of hypertension. *The Canadian journal of cardiology* 2015; **31**(5): 549-68.

9. Shimamoto K, Ando K, Fujita T, et al. The Japanese Society of Hypertension Guidelines for the Management of Hypertension (JSH 2014). *Hypertension research : official journal of the Japanese Society of Hypertension* 2014; **37**(4): 253-390.

10. Stergiou GS, Zourbaki AS, Skeva, II, Mountokalakis TD. White coat effect detected using self-monitoring of blood pressure at home: comparison with ambulatory blood pressure. *American journal of hypertension* 1998; **11**(7): 820-7.

11. O'Brien E, Parati G, Stergiou G. Ambulatory blood pressure measurement: what is the international consensus? *Hypertension* 2013; **62**(6): 988-94.

12. Pickering TG, Hall JE, Appel LJ, et al. Recommendations for blood pressure measurement in humans and experimental animals: Part 1: blood pressure measurement in humans: a statement for professionals from the Subcommittee of Professional and Public Education of the American Heart Association Council on High Blood Pressure Research. *Hypertension* 2005; **45**(1): 142-61.

13. Piper MA, Evans CV, Burda BU, Margolis KL, O'Connor E, Whitlock EP. Diagnostic and predictive accuracy of blood pressure screening methods with consideration of rescreening intervals: a systematic review for the U.S. Preventive Services Task Force. *Annals of internal medicine* 2015; **162**(3): 192-204.
